# Minimally Invasive Hepatectomy for Liver Tumors: Where Are We Now?

**DOI:** 10.3390/jcm12144583

**Published:** 2023-07-10

**Authors:** Kelvin K. C. Ng

**Affiliations:** 1 Department of Surgery, The Chinese University of Hong Kong, New Territories 852, Hong Kong; kkcng95@gmail.com; 2 Department of Surgery, Prince of Wales Hospital, New Territories 852, Hong Kong

With advancements in minimally invasive (MIS) technology and techniques, MIS hepatectomy has evolved as an effective treatment for both benign and malignant liver tumors. It has been increasingly utilized and studied extensively around the world. MIS hepatectomy bears all of the advantages of MIS surgery, i.e., smaller wounds, fewer wound and chest problems, a smaller risk of ileus, and quicker postoperative recovery. More importantly, it is associated with less surgical stress, less intraoperative blood loss, and less tumor manipulation during surgery. Potentially, it may have additional oncological benefits over the open approach. Nevertheless, MIS hepatectomy is technically challenging and a surgeon’s experience plays an important role in achieving a safe and successful operation. The first and second International Consensus Conferences on MIS hepatectomy have confirmed and outlined its clinical applications [1,2]. Currently, there are some areas of recent advancements in the field of MIS hepatectomy.

A crucial component in the success of MIS hepatectomy is ascertaining patients’ safety, which is influenced by short-term perioperative outcomes (the operative mortality and postoperative complications). Post-hepatectomy liver failure is considered to be the major hurdle of liver surgery. It is also an important predictor for mortality and morbidity following MIS hepatectomy. To prevent post-hepatectomy liver failure, an accurate preoperative assessment of the liver functional reserve is of utmost importance for the stringent patient selection for MIS hepatectomy. Traditionally, the Child–Pugh classification and indocyanine green (ICG) retention test have been adopted for the preoperative liver functional reserve assessment. Rahimli et al. [3] applied another novel test, the LiMAx test, for the preoperative liver function assessment in 40 patients who underwent MIS hepatectomy. The LiMAx test is based on the change in CO_2_ concentration measured via the breath test, which is caused by the metabolism of the intravenously applied ^13^C-methacetin. The cut-off value of the LiMAx test for normal liver function is >315 μg/h/kg. The study revealed that patients with a normal LiMAx test were more likely to undergo major hepatectomy, have a longer operation time, and require more intraoperative blood transfusions than those with a decreased LiMAx test. Hence, the LiMAx test is useful in precisely determining the liver function capacity. It helps select patients for major or minor MIS hepatectomy with regard to their safety. Apart from the liver functional reserve assessment, an accurate prediction model for postoperative complications following MIS hepatectomy is of equal clinical relevance. It has been shown that postoperative complications following hepatectomy had an adverse impact on patient survival outcomes and tumor recurrence in patients with malignant liver tumors [4]. Haber et al. [5] derived and validated the preoperative and postoperative models using clinical characteristics to predict postoperative complications in 210 patients with MIS hepatectomy. The preoperative model had five factors, including diabetes, a history of previous hepatectomy, the surgical approach, ALT levels, and the nature of the liver lesion. Meanwhile, two additional intraoperative factors (operative time and the need for conversion) were incorporated into the postoperative model. Both models were able to predict major postoperative complications after MIS hepatectomy with high accuracy (an area under the receiver operating characteristic curve > 0.7). Internal validation confirmed the discriminatory ability of the models. To facilitate the use of these models, a user-friendly online tool has been provided to clinicians to estimate the chance of postoperative complications without the need for a manual calculation (https://laprs.shinyapps.io/RiskScore/ (accessed on 1 July 2023)).

Since hepatocellular carcinoma (HCC) is the most common primary liver cancer in the world, much attention has been paid to the outcome of MIS hepatectomy for HCC. The latest published meta-analysis based on 45 propensity-score-matched studies found that MIS hepatectomy could improve recurrent-free survival in patients with HCC undergoing minor hepatectomy when compared to the open approach [6]. Nonetheless, data on MIS hepatectomy for other liver tumor types are lacking in the literature. Among these, intrahepatic cholangiocarcinoma (iCCA) has a unique identity since hepatectomy is the only potentially curative treatment with long-term survival benefits. Ratti et al. [7] reported on the clinical outcomes of MIS hepatectomy for iCCA in 179 patients. Compared with its open counterpart, MIS hepatectomy could achieve a similar number of retrieved lymph nodes, R0 resection rates, and negative resection margins. There were no differences between the MIS and open groups in overall survival, disease-free survival, and tumor recurrence rates. Nonetheless, the interval time between surgery and subsequent adjuvant treatments was significantly shorter in the MIS group than the open group. This is one of the merits of MIS hepatectomy which is associated with fewer postoperative complications and a shorter hospital stay. Thus, patients can receive adjuvant therapy on time, resulting in satisfactory oncological control of the tumor.

Precise and meticulous MIS techniques are key elements of MIS hepatectomy. Recently, ICG fluorescence navigation has been a hot topic in MIS hepatectomy. When injected intravenously, ICG is rapidly bound to plasma protein and remains in the bloodstream. It is taken up exclusively by hepatic parenchymal cells which excrete it into the bile. ICG has an emission peak of 835 nm, which allows for penetration of the tissue up to a depth of around 1 cm, and therefore visualization of tissue by an infrared camera using this dye is within 8 mm. Clinically, ICG is used for the intraoperative real-time visualization of bile ducts, the differentiation between cancerous and normal time, and the delineation of precise liver transection lines (positive vs. negative staining). There are international consensus statements on the use of fluorescence imaging in hepatobiliary surgery [8]. Mehdorn et al. [9] evaluated the use of ICG in MIS hepatectomy, which was assisted by a robotic approach in 54 patients. ICG-guided MIS hepatectomy had a shorter operating time and higher R0 rate than that without ICG navigation. This illustrates the advantages of utilizing ICG fluorescence navigation in MIS hepatectomy in terms of a safe and effective surgery, and a high rate of oncological tumor clearance. Similar results were also obtained in another study on 120 patients who underwent MIS hepatectomy [10]. Apart from ICG fluorescence navigation, three special device techniques for use in the liver transection of an MIS hepatectomy were advocated for by Perrakis et al. [11] Using these techniques, the precise intrahepatic control of blood vessels and bile ducts could be effectively achieved. There was a 100% R0 resection rate, 10.7% complication rate, and 0% mortality rate when using these techniques.

In MIS hepatectomy, there is a well-established learning curve in terms of optimizing conversion rates, operative times, blood loss, and complication rates. A study utilized the cumulative sum analysis on 174 patients who underwent MIS hepatectomy and determined the learning curve to be 60 cases [12]. In addition to the hands-on training in the operating theater, virtual reality simulator training is of equal importance. Lefor et al. [13] developed and validated a novel virtual reality simulator for MIS hepatectomy. It was coded in C++ using PhysX and FleX with a novel cutting algorithm and used a patient data-derived model and two instruments functioning as ultrasonic shears. This system was tested by experts and novices. It had high scores for the simulator interface, the appropriateness for education, the effectiveness of the interface, motion consistency, and realistic soft tissue behavior.

The usual approaches (laparoscopic and robotic-assisted approaches) of MIS hepatectomy have been widely studied in the literature. Graur et al. [14] performed a systemic review on another two novel approaches (single-incision laparoscopic surgery (SILS) and natural orifice transluminal endoscopic surgery (NOTES)). The mean procedure time was up to 205 min, and the mean diameter of liver lesions was 5 cm. The authors concluded that SILS and NOTES were less intrusive, more easily tolerated, and more esthetic. 

Up till now, there are still many areas of MIS hepatectomy that are being developed and studied widely. Whether MIS hepatectomy can replace conventional open hepatectomy in most cases is believed to be not just a dream, but eventually will be a reality.
